# In this Issue – 8.5

**DOI:** 10.1080/17571472.2016.1226268

**Published:** 2016-09-20

**Authors:** Paul Thomas

This Issue of LJPC displays the breadth of matters that primary care practitioners routinely care about.

Howells, McKay, Hussein and Majeed provide guidance on how to manage people who are at high risk of developing diabetes. ‘Intermediate’, or ‘non-diabetic hyperglycaemia’ are those whose HbA1c level is between 42 and 47 mmol/mol (6.0–6.4%) or have fasting blood glucose measurement of 5.5–6.9 mM. They need to be monitored.

Thorne examines ways that the quality and outcomes framework could do more to tackle health inequalities. This is a hot topic, exposed by the BMJ in August 2016. Thorne suggests ways to address the challenge.

Ma and Shah discuss the shortage of training places and long waiting list for clinicians who wish to train in sexual and reproductive healthcare. Their survey of medical educators identified considerable numbers of GP already trained and interested in having such roles. They signal a need for us all to be aware of the untapped potential sitting under our noses.

In a comparison of two legal cases that arrived at exactly opposite conclusions, Gee elegantly describes how to think about doing the right thing by a patient who has lost mental capacity. It is important to weigh up the wishes, feelings, beliefs and values of a mentally incapacitated patient when deciding what treatment to give. This paper is worth reading simply to see the quality of thinking of our judges as well as how we make decisions about patient care.

Through the Hippokrates Exchange Program, Siraj shadowed an experienced GP in Madrid. She found considerable similarities with what a GP does in the UK and some interestingly different things, including an intense working regime (7 min with a patient) and excellent continuity of care. Siraj makes the point that the paternalistic consultation style, less favoured these days, can be effective when applied appropriately and respectfully, but it does increase referral rates!

Paul Thomas Paul.thomas7@nhs.net

